# Interferon mediated neuroinflammation in polyglutamine disease is not caused by RNA toxicity

**DOI:** 10.1038/s41419-019-2193-x

**Published:** 2020-01-02

**Authors:** Aksheev Bhambri, Akeeth Pinto, Beena Pillai

**Affiliations:** 1 Academy of Scientific and Innovative Research (AcSIR), New Delhi, India; 2 Institute of Genomics and Integrative Biology (IGIB), Mathura Road, New Delhi, India

**Keywords:** Cell death in the nervous system, Huntington's disease

## Abstract

Polyglutamine diseases are neurodegenerative diseases that occur due to the expansion of CAG repeat regions in coding sequences of genes. Previously, we have shown the formation of large protein aggregates along with activation of the interferon pathway leading to apoptosis in a cellular model of SCA17. Here, we corroborate our previous results in a tetracycline-inducible model of SCA17. Interferon gamma and lambda were upregulated in 59Q-TBP expressing cells as compared to 16Q-TBP expressing cells. Besides interferon-stimulated genes, the SCA17 model and Huntington’s mice brain samples showed upregulation of RNA sensors. However, in this improved model interferon pathway activation and apoptosis preceded the formation of large polyglutamine aggregates, suggesting a role for CAG repeat RNA or soluble protein aggregates. A polyglutamine minus mutant of TBP, expressing polyCAG mRNA, was created by site directed mutagenesis of 10 potential start codons. Neither this long CAG embedded mRNA nor short polyCAG RNA could induce interferon pathway genes or cause apoptosis. polyQ-TBP induced the expression of canonical RNA sensors but the downstream transcription factor, IRF3, showed a muted response. We found that expanded CAG repeat RNA is not sufficient to account for the neuronal apoptosis. Neuronal cells sense expanded CAG repeats embedded in messenger RNAs of protein-coding genes. However, polyglutamine containing protein is responsible for the interferon-mediated neuroinflammation and cell death seen in polyglutamine disease. Thus, we delineate the inflammatory role of CAG repeats in the mRNA from the resulting polyglutamine tract in the protein. Embedded in messenger RNAs of protein-coding regions, the cell senses CAG repeat expansion and induces the expression of RNA sensors and interferon-stimulated genes.

## Introduction

The mammalian genome is interspersed with polymorphic repeats present in geneic and non-geneic regions. Trinucleotide repeats when present in the appropriate coding frame can lead to the formation of homopolymeric stretches in the resulting proteins. CAG repeats in the coding region of the gene, for instance, code for polyglutamine (polyQ) tracts in the corresponding protein. This polyQ tract may even be functional and required by the protein^1,2^. However, due to the repetitive nature of the polyQ coding region, it is prone to DNA slippage leading to expansion of the CAG repeats^3^. In 1–10 per 100,000 cases^4^, when the CAG repeats expand beyond a certain threshold, it leads to aggregation of the protein leading to a dominant neurodegenerative disease. This group of nine diseases are called polyglutamine diseases. Among the polyQ diseases, Huntington’s disease (HD) which has a prevalence of 5.96–13.7 per 100,000^5^, is the most well studied. Although they have been studied separately, many mechanisms of apoptosis such as autophagy^6^, unfolded protein response^7^ and mitochondrial dysfunction^8–10^ have been implicated in multiple polyglutamine diseases.

One such mechanism implicated in multiple polyQ diseases, through several lines of evidence, is RNA mediated toxicity. In Drosophila, CAG tracts interspersed with the degenerate codon, CAA is associated with less severe phenotype^11^. The same effect was also seen in human patients^12,13^ with a recent report showing up to 25 year reduction in age of onset due to the loss of CAA interruptions in HD patients^14^. This is important as CAG repeat toxicity is often linked to its hairpin structure seen in vitro, which is disrupted when it is interspersed with CAA^15^. It has been proposed that this hairpin structure may be recognized by the RNAi machinery leading to the formation of small CAG RNAs that target genes containing complementary CTG repeats in their untranslated regions (UTRs)^16^. Moreover, the CAG repeat RNA sequesters proteins preventing them from performing their designated function. CAG repeat RNA have been shown to sequester MBNL1^17^, nucleolin^18^, and Protein Kinase R (PKR)^19^. Although the precise mechanism and extent of CAG RNA toxicity is not firmly established, several lines of evidence indicate its potential to modulate disease phenotypes. In diseases like Spinocerebellar Ataxia 12 (SCA12), that are, like polyQ diseases, marked by tremor and cerebellar atrophy, the CAG expansion is in the untranslated regions (UTRs) of the messenger RNA. CAG repeat in the UTR of marker proteins such as EGFP also have been shown to cause apoptosis^20^. Antisense oligonucleotides (ASO) that reduce the RNA but not the huntingtin protein reduced the severity of the phenotype^21^.

Among the nine polyglutamine disease, Spinocerebellar Ataxia 17 (SCA17) has the least prevalence of 0.47 per 1,000,000, as reported in Japanese population^22^. SCA17 occurs due to CAG repeat expansion in the TATA-box binding protein (TBP), a ubiquitous general transcription factor. An expansion beyond 43–45 glutamines in TBP makes it aggregation prone leading to late onset neurodegeneration^23^. Although the prevalence is low, SCA17 shows high phenotypic similarity to HD, so much so that it was earlier named HD-like 4 (HDL 4)^23^. Moreover, in most of the polyQ diseases, transcriptional dysregulation is seen and TBP is one of the protein sequestered by the aggregating polyQ protein^23^. Hence, studying SCA17 could potentially reveal common pathways in polyQ diseases.

Our lab has been using a cellular model of SCA17 in which we have shown the upregulation of VDAC1 leading to apoptosis^24^. Moreover, we have shown that miR-29a/b targets VDAC1 as well as BACE1, preventing apoptosis in the neuronal cells^25^ and ataxia in mice^26^. Recently, we showed that interferon signaling is elevated in this cellular model of SCA17, prompting the hypothesis that intercellular signaling leading to bystander cell death may aggravate the pathology of the disease^27^. Here, we explored the response of neuronal cells to CAG repeat RNA, specifically testing if CAG repeat RNA mediated toxicity was sufficient to explain the aberrant interferon signaling and cell death seen in polyQ expressing neuronal cells. We show that CAG repeat RNA devoid of the protein-coding ability is not toxic. Further, a mutant TBP expressing a N-terminal truncated TBP devoid of polyglutamine was not toxic inspite of the mRNA containing an interrupted polyCAG (93nt) stretch. Although canonical RNA sensor proteins were upregulated in response to the CAG repeat RNA embedded in mRNA, it did not lead to aberrant interferon signaling.

## Materials and methods

### Cell culture

Neuro2A cell line was cultured in Dulbecco’s Modified Eagle’s Media (DMEM) (Invitrogen) supplemented with 10% fetal bovine serum (Invitrogen) and incubated in a humidified incubator with 5% CO_2_. Tetracycline-inducible stable lines were cultured in DMEM supplemented with 10% tetracycline-reduced fetal bovine serum (Clonetech).

### Generation of tetracycline-inducible cell lines

Human TATA-box binding protein (TBP) gene with 16Q or 59Q repeats was cloned in pCW57.1 (addgene #41393) as fusion proteins with EeGFP. This construct was then transfected with packaging vectors, pCMV-VSV-G and pCMV-dR8.2 (Gifted by Dr. Rajendra Motiani, CSIR IGIB) into Human Embryonic Kidney (HEK) 293T LentiX cell line (Gifted by Dr. Rajendra Motiani, CSIR IGIB) using Xfect^TM^ Transfection Reagent (Clonetech). The media was collected 48 hr after transfection and was filtered through a 0.45 µm filter. This was used to transduce Neuro2A cells with 8 µg/ml Hexadimethrine bromide (Sigma). Puromycin (Sigma) selection was performed 48 hr after media treatment. Selected cells were expanded and then serially diluted to get single clones in a 48well plate. Clones were screened for optimum expression of the insert and one cell line each for 16Q-TBP and 59Q-TBP was selected for further experiments.

### Cytochrome c assay

16Q-TBP and 59Q-TBP cells were collected 48 h after induction using 5 mM sodium butyrate (Sigma) and 6 µg/ml doxycycline (Clonetech). Cells were resuspended in extraction buffer (10 mM HEPES, pH 7.5, 200 mM mannitol, 70 mM sucrose, 1 mM EGTA and 2 mg/ml bovine serum albumin) and incubated for 1 h on ice. The resuspended cells were then lysed using type-B Dounce homogenizer (Sigma). The lysate was spun down at 13,000 rpm for 10 min at 4° C. The supernatant was collected and used for cytochrome c assay using Rat/Mouse Cytochrome c Quantikine ELISA kit (R&D Systems, MCTC0).

### Quantitative reverse transcriptase-PCR(qRT-PCR) and immunoblotting

16Q-TBP and 59Q-TBP cells were induced using 6 µg/ml doxycycline (Clonetech) and 5 mM sodium butyrate (Sigma). Cells were harvested 48 h after treatment using TRIzol reagent (Invitrogen) for RNA isolation. RNA was isolated as per the manufacturer’s protocol. cDNA synthesis was performed using M-MuLV Reverse Transcriptase (NEB) and quantitative PCR (qPCR) was performed using KAPA SYBR Master Mix. Primers used in the study have been listed in Supplementary Table 1. For protein extraction, induced cells were harvested 48 hr after treatment using RIPA buffer (Sigma). Protein was quantitated using BCA method and Immunoblotting was performed. Primary antibodies for STAT1 (Cell Signaling Technologies, 9172), pSTAT1 (Cell Signaling Technologies, 7649), TBK1 (Abcam, ab40676) and IRF3 (Cell Signaling Technologies, 4302) were used at 1:1000 dilution. Anti-polyglutamine-expansion specific antibody (Millipore, MAB1574), Anti-GFP (Abcam, ab290) and GAPDH (Santa Cruz Biotechnology, sc32233) were used at 1:5000 dilution. was used a housekeeping control at 1:5000 dilution. Secondary antibodies used was conjugated with Horseradish Peroxidase or by infrared dye and hence, blots were developed using chemiluminescence or scanned in infrared LI-COR Odyssey scanner, respectively.

### Immunofluorescence and confocal imaging

The induced cells were fixed 48 h after induction using 4% para-formaldehyde. For MG132 experiment, 16Q-TBP and 59Q-TBP cells were induced for 24 h after which 10 µM MG132 was added to the cells. DMSO of the same volume was added for vehicle control. Cells were fixed 24 h after MG132 or DMSO treatment using 4% para-formaldehyde. Fixed cells were washed with PBS and blocked using 5% goat serum in PBS with 0.3% Triton X-100. Primary antibodies used were Anti-GFP (Abcam, ab290) at 1:5000. Secondary antibody used was Anti-Rabbit conjugated with Alexa Fluor 488 (Molecular Probes) at 1:1000 dilution. DAPI (Sigma) was used for staining the nuclei and Slow Fade Mountant (Invitrogen) for mounting coverslips on the slides. Imaging was performed using Leica SP8 confocal microscope.

### Transfection experiments

pcDNA3.1 vectors was used to clone 16CAG repeats and 59CAG repeats from 16Q-TBP and 59Q-TBP vectors used in the previous studies^24^. Briefly, regions adjacent to CAG repeats were used to design primers, 5’-GGAAGAGCAACAAAGGCAGCAG-3’ and 5’-ACGGCTGCAGCTGCCACTG-3’. PCR was performed and fragments were cloned in linearized pcDNA3.1 vector. Clones were confirmed by sanger sequencing. pEGFP-N3 vector was used for cloning of PolyQ^-^ TBP mutant. The region of the gene to the 5’-end of the gene was made by overlapping PCR using the primers: 5’-ATATCTCGAGATCGATCAGAACAACAGCCTCCCACCTTACGCTCAGGGCTTCGCCTCCCCTCAGGGTGCCATCACTCCCGGAATCCC-3’, 5’-ATAGGCTGTGGGGTGAGTCCAGTGCGATAAGGGATGATTGGACTAAAGATAGGGATTCCGGGAGTGATGGCACCCTGAGG-3’, 5’-CTGCTGCCTTTGTTGCTCTTCGAAAATAGAGAGACTATTGGTGTTCTGAATAGGCTGTGGGGTGAGTCCAGT-3’. The final product was annealed onto the 59Q-TBP gene and extension was performed. This product was reamplified using forward and reverse primers: 5’-ATATGGATCCCGTCGTCTTCCTGAATCCCT-3’, 5’-ATATCTCGAGATCGATCAGAACAACAGCCTC-3’. The final product and pEGFP-N3 was digested with BamHI and XhoI. Ligation was performed and the transformation was performed. Clones were confirmed by sanger sequencing. The pcDNA3.1-16CAG and pcDNA3.1-59CAG plasmids were transfected separately in Neuro2A cells using Lipofectamine LTX (Invitrogen). Quantitative PCR and immunoblotting were performed as described before.

### Huntington mice experiments

B6CBA-R6/2 (CAG120+/−5) mice and age matched control mice were dissected at the age of 84 days. Cortex and cerebellum were dissected and frozen in liquid nitrogen. The samples were kept in −80° C freezer until they were used for RNA and protein extraction as described before. These samples were a kind gift from Dr. Nihar Ranjan Jana, National Brain Research Centre, India.

## Results

We have previously shown that the polyglutamine-TBP-GFP bearing a pathogenic CAG repeat tract of 59 caused apoptosis in neuronal cell lines^24^. In this model, the protein was constitutively expressed leading to the formation of large nuclear aggregates. However, the dynamics of protein aggregation or the concomitant toxicity could not be studied in such a constitutive model. Moreover, transient transfection of the plasmids resulted in heterogeneity in the cells. We, therefore, created stable derivatives of Neuro2A wherein the normal 16Q-TBP-GFP or the pathogenic 59Q-TBP-GFP could be rapidly expressed under the tight control of a tetracycline-inducible promoter.

**Fig. 1:**
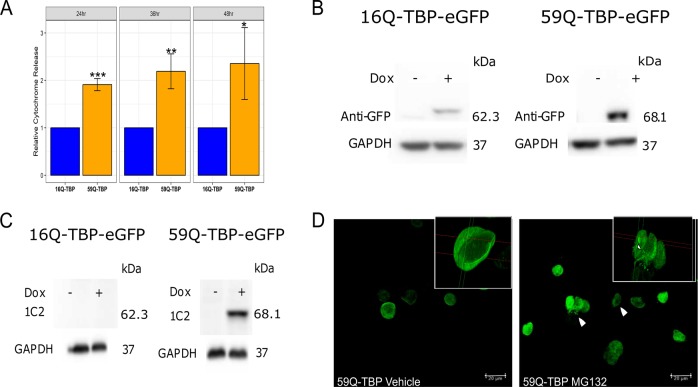
Tetracycline-inducible stable cell lines for SCA17 recapitulate polyQ-TBP toxicity. **a** Cytochrome c released in the cytosol measured in 16Q-TBP-GFP and 59Q-TBP-GFP cell lines 24, 36, and 48 h after induction. *n* = 3. **b** Immunoblotting performed using anti-GFP antibody and **c** anti-polyglutamine (1C2) antibody immunoblot using uninduced and induced 16Q-TBP-GFP and 59Q-TBP-GFP cells. **d** Confocal images of vehicle (DMSO) and MG132 treated 59Q-TBP induced cells. White arrow shows protein aggregates seen as puncta in the nucleus on MG132 treatment. Inset shows 3-dimensional projection of the nucleus depicting aggregates by white arrows. Students *t*-test was performed. *p-value ≤ 0.05; **p-value ≤ 0.01; ***p-value ≤ 0.001.

Doxycycline treatment of 24 h was sufficient to trigger a 2-fold increase in neuronal apoptosis (Fig. 1a). Sustained expression of the toxic protein did not cause a further increase in cell death as measured by cytochrome c release into the cytosol (Fig. 1a). We also monitored the inducible expression of 16Q-TBP-GFP and 59Q-TBP-GFP at 48 h after doxycycline treatment by immunoblotting with antibody against the GFP tag (Fig. 1b) as well as the 1C2 antibody against expanded polyQ (Fig. 1c). In agreement with our previous findings, the 1C2 antibody detected the 59Q-TBP protein but not the 16Q-TBP. The absence of a detectable band in uninduced conditions rules out any leaky expression. A striking difference between the constitutive and inducible models was the pattern of aggregation. Induction of the polyQ protein for even 48 h did not produce large aggregates that are readily seen in 36 h of constitutive expression. Notably, apoptosis is induced well before and in the absence of large aggregates suggesting that polyCAG RNA or misfolded intermediates may be causative agents in triggering cell death. To verify that the inducible 59Q-TBP-GFP could form aggregates, we treated these cells with MG132, an inhibitor of proteasomal machinery. Within 24 h of treatment, large nuclear protein aggregates were formed (Fig. 1d).

**Fig. 2:**
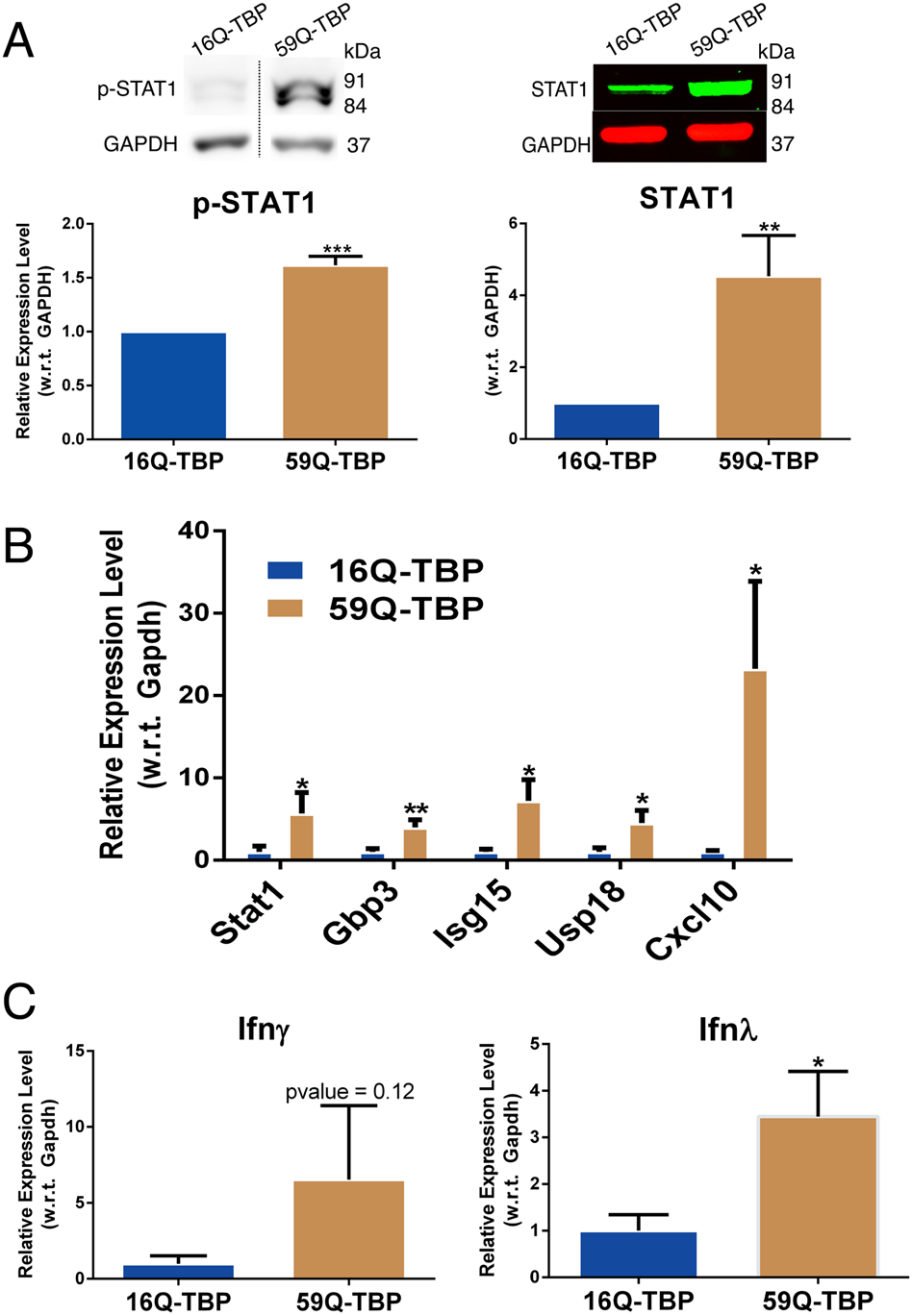
Type-II and Type-III interferons are expressed in tetracycline-inducible SCA17 model leading to activation of interferon pathway. **a** Immunoblotting performed using anti-phosphorylated STAT1 and anti-STAT1 antibodies on induced 16Q-TBP-GFP and 59Q-TBP-GFP cells. *n* = 3. Top panel: western blot; bottom panel: quantitation of the bands shown in top panel) **b** quantitative RT-PCR performed for interferon-stimulated genes on 16Q-TBP-GFP and 59Q-TBP-GFP cells. *n* = 3. **c** quantitative RT-PCR performed for interferon genes on 16Q-TBP-GFP and 59Q-TBP-GFP cells. *n* = 3. Students *t*-test was performed. *p-value ≤ 0.05; **p-value ≤ 0.01; ***p-value ≤ 0.001.

We have previously shown that neuronal cells expressing toxic 59Q-TBP protein induce aberrant interferon signaling resulting in Stat1 mediated upregulation of interferon-stimulated genes (ISGs)^27^. In the tetracycline-induced cell lines used in the study, we verified that STAT1 protein is induced and activated by phosphorylation (Fig. 2a). A panel of ISGs (Gbp3, Isg15, Usp18, and Cxcl10) were transcriptionally induced along with Stat1 mRNA, in agreement with previous findings (Fig. 2b). Viruses, misfolded proteins, and bacterial antigens trigger interferon release, a mechanism for cell-to-cell signaling during cellular stress. Cell type specific expression of receptors to type I and III interferon modulate the response but eventually leads to induction of a common set of ISGs^28^. Hence, these ISGs are not very informative about which interferon type may be involved. Interferon secreted by neuronal cells was below the detection limit of currently available methods, thus preventing direct detection of secreted interferon. Therefore, we used mRNA levels as a substitute for finding which interferon may be involved. While interferon beta gene expression was not detected, the large number of genes that code for interferon alpha posed technical issues in qRT-PCR based detection. However, interferon gamma and the more recently discovered interferon lambda were transcriptionally induced in 59Q-TBP-GFP expressing cells as compared to 16Q-TBP-GFP expressing cells (Fig. 2c).

**Fig. 3:**
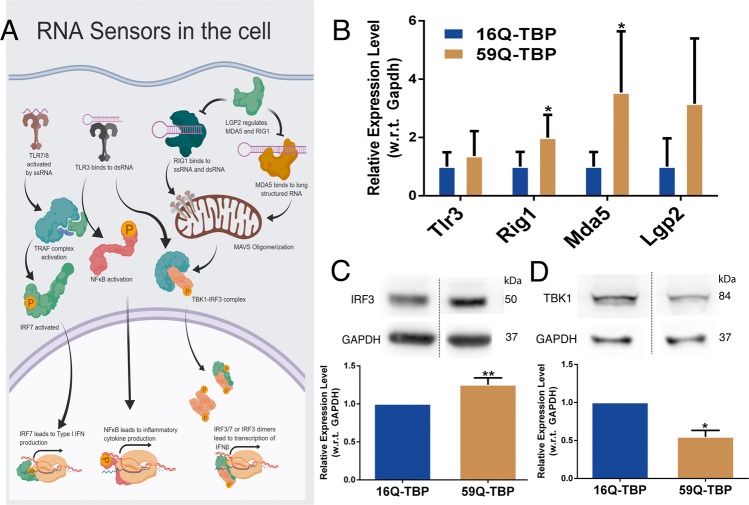
RNA sensors are upregulated in SCA17 cellular model. **a** Schematic representation showing RNA sensors in the cell (made in Biorender). **b** Quantitative RT-PCR for RNA sensors performed in 16Q-TBP-GFP and 59Q-TBP-GFP cells. *n* = 3. **c** Immunoblotting performed for IRF3 protein on 16Q-TBP-GFP and 59Q-TBP-GFP cells. *n* = 3. **d** Immunoblotting for TBK1 protein in 16Q-TBP-GFP and 59Q-TBP-GFP cells. *n* = 2. Students *t*-test was performed. *p-value ≤ 0.05; **p-value ≤ 0.01; ***p-value ≤ 0.001.

As shown in Fig. 1, apoptosis preceded the formation of large protein aggregates suggesting a possible role for RNA toxicity. The interferon pathway is known to be induced in response to double-stranded RNA (dsRNA) intermediates formed during viral infections. Poly-CAG repeats in the mRNA could activate interferon pathway by forming stem loop structures. We monitored the expression of a variety of RNA sensors that are known to bind to viral RNA (Fig. 3a, b). Rig1, the major intracellular RNA sensor, recognizes free 5’ termini while Mda5 binds to higher order structures formed by long transcripts^29,30^. Interestingly, we found RIG1 to be modestly upregulated (2.2-fold) while Mda5 was highly induced (3.6 fold) (Fig. 3b). When we studied the expression of IRF3, the downstream transcription factor, its upregulation was merely 1.3fold (Fig. 3c). This prompted us to examine the levels of Tbk1, the protein that forms a complex with a variety of proteins ultimately resulting in activation of IRF3^31^, and Lgp2, a negative regulator of Rig1^32,33^. In keeping with the muted IRF3 response, we find that Tbk1 is downregulated and Lgp2 is robustly induced (4.2-fold) (Fig. 3b, d).

**Fig. 4:**
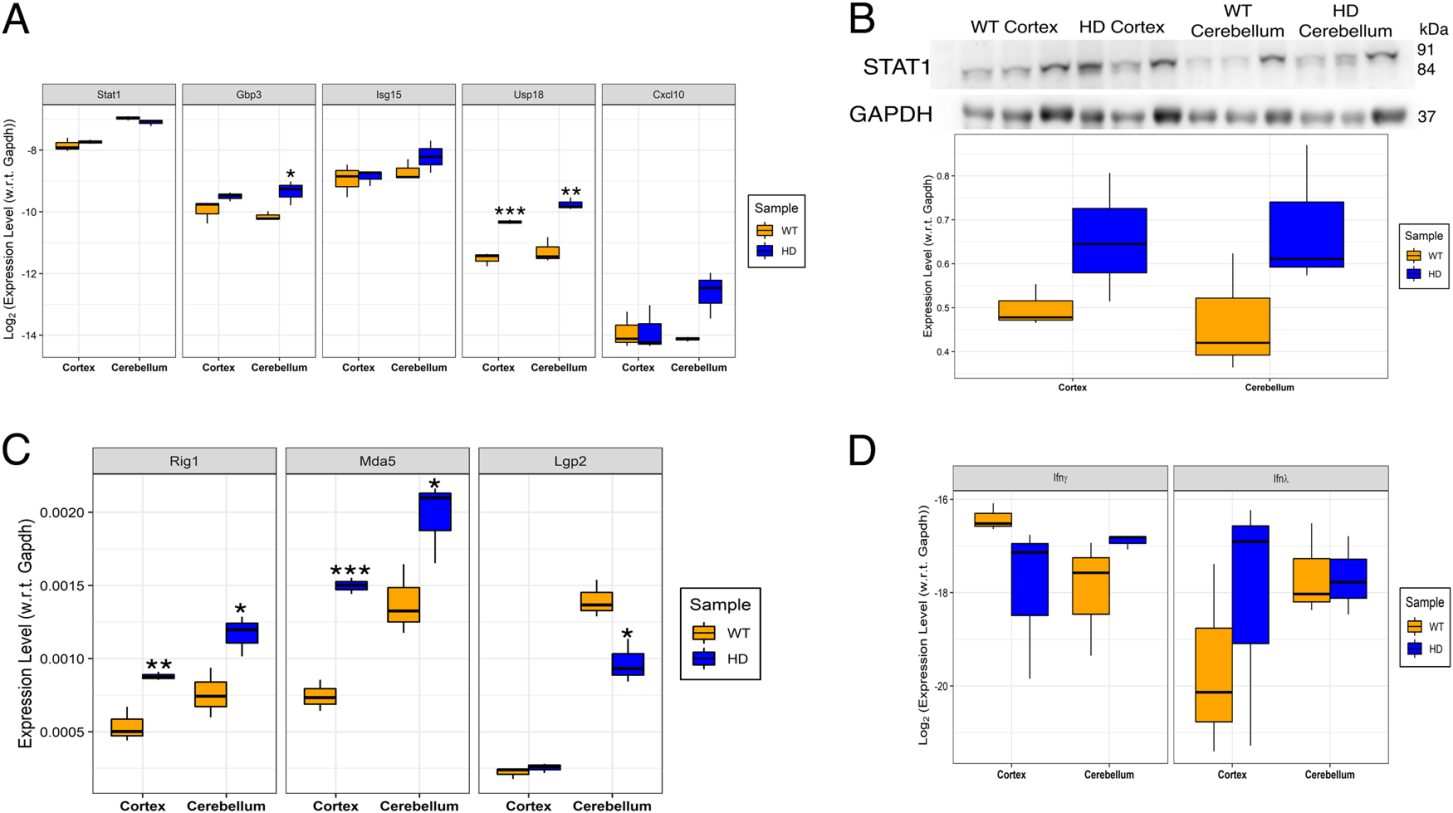
RNA sensors and interferon-stimulated genes are upregulated in Huntington’s disease. **a** Quantitative RT-PCR performed for interferon-stimulated genes on cortex and cerebellum isolated from wild type (WT) and Huntington’s mice (HD). **b** Immunoblotting performed using anti-STAT1 antibody on cortex and cerebellum isolated from wild type (WT) and Huntington’s mice (HD). **c** Quantitative RT-PCR performed for RNA sensors (Rig1, Mda5, Lgp2) on cortex and cerebellum isolated from wild type (WT) and Huntington’s mice (HD). *n* = 3. **d** quantitative RT-PCR performed for interferon genes in cortex and cerebellum isolated from wild type (WT) and Huntington’s mice (HD). Students *t*-test was performed. *p-value ≤ 0.05; **p-value ≤ 0.01; ***p-value ≤ 0.001.

Besides SCA17, interferon signaling could be relevant in other polyQ diseases. We hypothesized that the RNA sensors and the interferon pathway could be upregulated in HD mice. ISGs were upregulated with Gbp3 in the cerebellum while Usp18 was upregulated in both cortex and cerebellum of HD mice compared to wild-type (WT) mice (Fig. 4a). Immunoblotting showed an increase in STAT1 protein in HD mice but it was not statistically significant (Fig. 4b). In agreement with our hypothesis, the canonical RNA sensors Rig1 and Mda5 showed stark upregulation in HD mice cortex and cerebellum as compared to wild type mice (Fig. 4c). Moreover, Lgp2, a regulator of Rig1 and Mda5, was significantly downregulated in HD mice cerebellum (Fig. 4c). However, expression of neither interferon gamma nor interferon lambda mRNA was elevated in HD mice as compared to wild type mice (Fig. 4d). Thus, we conclude that although CAG repeat RNA produces dsRNA intermediates that are sensed by canonical RNA sensors, downstream modulation prevents the signal from triggering a classical interferon response.

**Fig. 5:**
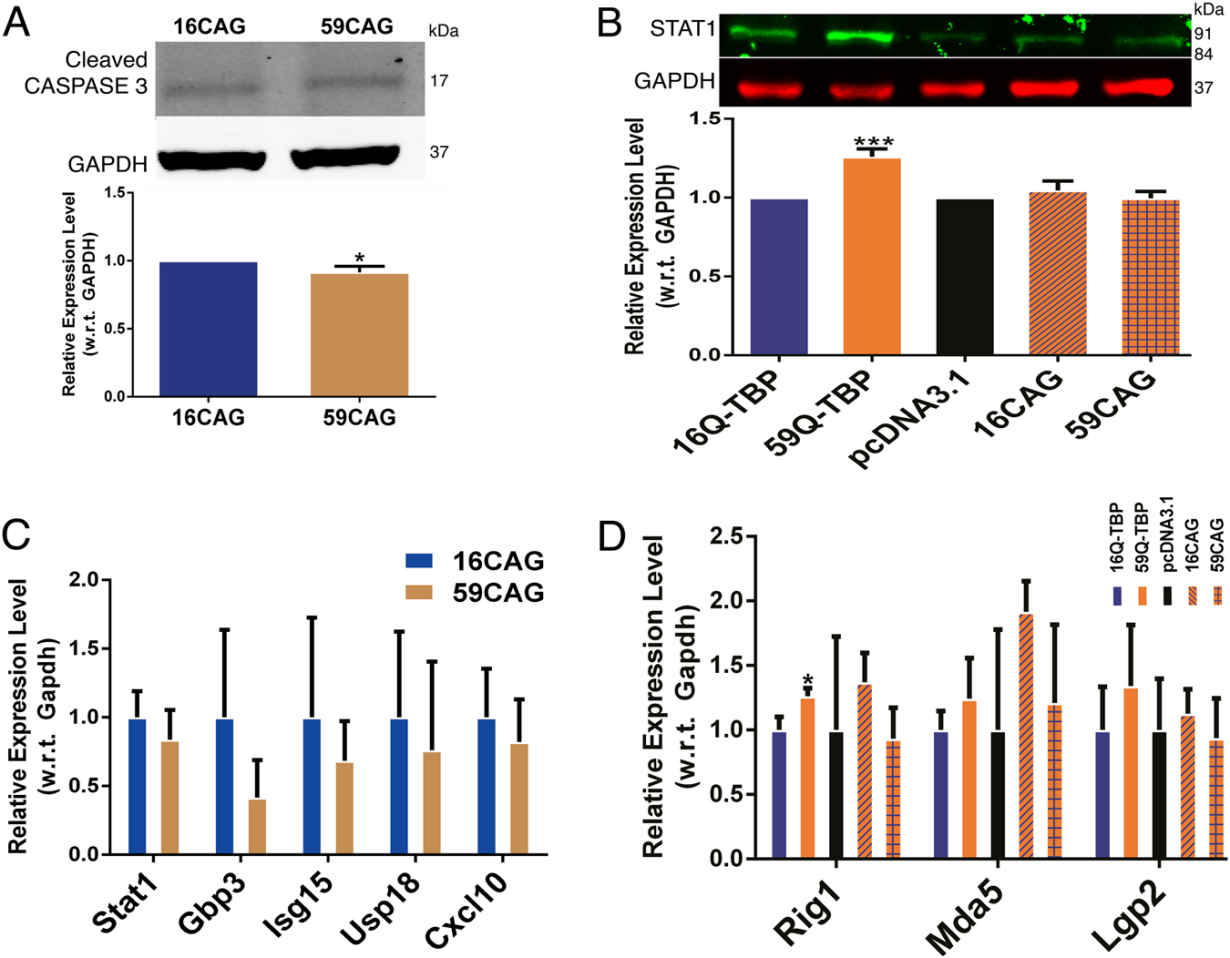
CAG repeat RNA alone can neither activate interferon pathway nor is it toxic to neuronal cells. **a** Immunoblotting performed for cleaved caspase 3 on pcDNA3.1-16CAG and pcDNA3.1-59CAG transfected cells. **b** Immunoblotting performed using anti-STAT1 antibody on 16Q-TBP-GFP, 59Q-TBP-GFP, pcDNA3.1, pcDNA3.1-16CAG and pcDNA3.1-59CAG transfected cells. **c** Quantitative RT-PCR performed for interferon-stimulated genes on pcDNA3.1-16CAG and pcDNA3.1-59CAG transfected cells. **d** Quantitative RT-PCR performed for RNA sensors (Rig1, Mda5, and Lgp2) on 16Q-TBP-GFP, 59Q-TBP-GFP, pcDNA3.1, pcDNA3.1-16CAG, and pcDNA3.1-59CAG transfected cells. *n* = 3. Students *t*-test was performed. *p-value ≤ 0.05; **p-value ≤ 0.01; ***p-value ≤ 0.001.

Next, we examined if the CAG repeat region alone was sufficient to cause upregulation of interferon-stimulated genes and cell death. We cloned 16CAG repeat and 59CAG repeats under the control of CMV promoter but devoid of TBP and GFP. The expression of CAG repeat RNA was verified by RT-PCR using primers against the minimal flanking regions (Supplementary Fig. 1). In this context, 16CAG and 59CAG transcripts resulted in similar levels of apoptosis as measured by caspase 3 cleavage (Fig. 5a). In agreement, there was no upregulation of STAT1 protein (Fig. 5b) and mRNA levels of ISGs (Fig. 5c) suggesting that the SCA17-derived CAG repeat is toxic only when embedded in the TBP-GFP context. Moreover, no upregulation of the canonical RNA sensors was observed in cells expression 16CAG or 59CAG RNA (Fig. 5d).

**Fig. 6:**
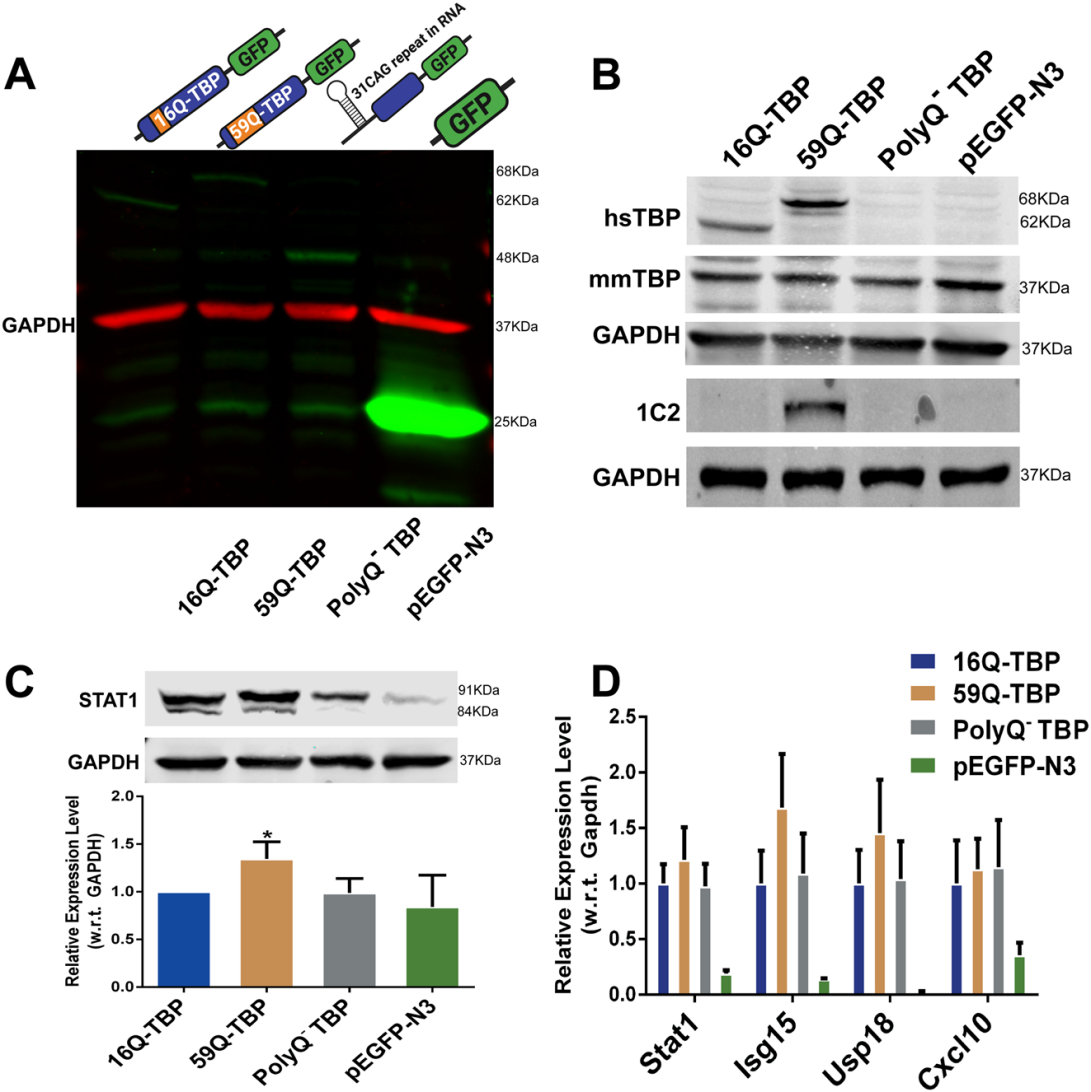
Mutant TBP construct expressing CAG repeat RNA but not polyQ protein cannot activate interferon pathway. **a** Immunoblotting performed using anti-GFP antibody, **b** anti-TBP antibody, anti-polyglutamine (1C2) antibody and **c** anti-STAT1 antibody on 16Q-TBP-GFP, 59Q-TBP-GFP, PolyQ^−^ TBP and pEGFP-N3 transfected neuronal cells at 36 hr post-transfection. **d** quantitative RT-PCR performed for interferon-stimulated genes on 16Q-TBP-GFP, 59Q-TBP-GFP, PolyQ^−^ TBP and pEGFP-N3 transfected neuronal cells at 36 h post-transfection. *n* = 3. Students was *t*-test performed. *p-value ≤ 0.05; **p-value ≤ 0.01; ***p-value ≤ 0.001.

The short CAG repeat transcript may be excluded from the biogenesis of a typical mRNA. To delineate the contribution of the CAG repeat mRNA and polyQ containing TBP protein, we created a mutant (Supplementary Fig. 2) that expresses TBP-GFP fusion mRNA (Fig. 6a) with 10 mutations that replace start codons (NTG) with NTC. The substitution of start codons was designed to produce an N-terminal truncated protein devoid of polyglutamine from an mRNA in spite of containing a part of the CAG repeat (93nt corresponding to a stretch of 31 glutamines). We verified that the resulting protein was detectable by western blotting using anti-GFP antibody (Fig. 6a) but not by the polyQ-specific 1C2 antibody (Fig. 6b). This mutant failed to induce Stat1 or Stat1-dependent genes while 59Q-TBP-GFP expressing cells showed elevated Stat1 protein, Stat1-dependent genes (Fig. 6c, d). Thus, we are able to show conclusively that the interferon-mediated neuroinflammation and cell death seen in the in vitro model of SCA17 is not caused by short, non-coding CAG RNAs.

## Discussion

Metanalysis of publicly available transcriptomic data showed that interferon-stimulated genes are upregulated in a number of neurodegenerative diseases besides SCA17 (Table 1). This suggests that a deeper understanding of common factors that trigger interferon signaling in neurodegeneration are needed. Several lines of evidence indicate that CAG repeats, a common feature of these diseases, may manifest their neuronal toxicity through RNA^14,16,18,20,34^. Although the spinocerebellar ataxias are largely caused by CAG repeat expansions in coding regions, several diseases like SCA12 have CAG repeat expansions in untranslated regions. Further, CXG (CUG in Myotonic Dystrophy; CCG/CGG in Fragile X syndrome) also lead to neuronal toxicity although they do not form polyglutamine. Irrespective of the nucleotide at the second position, CXG tracts are capable of folding back upon themselves to form hairpin structures^35^. It has been suggested that these hairpin structures can be processed by double-strand RNAases in the cell to give rise to toxic dsRNA intermediates^16^. Mammalian cells have developed a pathway to detect and respond to viral dsRNAs as a form of innate immunity^29^. This study was undertaken to test the specific hypothesis that CAG repeat derived dsRNA may mimic viral dsRNA response leading to interferon-mediated cell death.

**Table 1:**
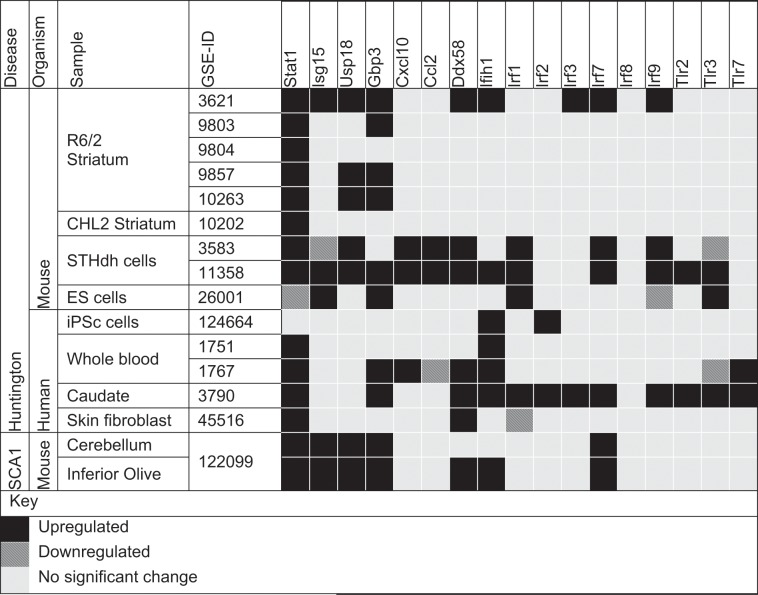
Interferon-stimulated genes and RNA sensors are upregulated in polyglutamine diseases: meta-analysis of Gene Expression Omnibus (GEO).

Our previous results showed that CAG repeat expansion in TATA-box binding protein leads to induction of interferon-stimulated genes and apoptosis^27^. Overcoming the limitations imposed by the constitutive expression of polyQ-TBP in our previous studies by doxycycline mediated induction of polyQ-TBP, we found that apoptosis preceded the formation of large protein aggregates. Therefore, we explored the possibility that dsRNA intermediates arising from the CAG repeat region were sufficient to trigger an interferon response and apoptosis.

We expressed a mutant TBP gene containing 93nt CAG repeats preceding the functional start codon, such that the mRNA could form CAG hairpin structures, but the translated protein would be devoid of polyQ. Alternatively, CAG repeat derived short ncRNA were expressed in neuronal cells. CAG repeats expressed as non-coding RNA or embedded within mRNA were neither sufficient to induce interferon-stimulated genes nor to cause cell death. Although polyQ-TBP-GFP induction led to upregulation of canonical RNA sensors, RIG1 and Mda5, a strong induction of IRF3 was missing. In spite of the coordinated upregulation of multiple RNA sensors, IRF3 upregulation is muted suggesting that there are internal regulatory mechanisms to contain the response to toxic RNA intermediates. Our observations suggest that TBK1, one of the activators of IRF3^31^, may modulate the signals from the RNA sensors before they are transmitted to the downstream transcriptional activator IRF3. This is in agreement with recent reports where isoforms of TBK1 are shown to inhibit type I interferon signaling in zebrafish^36^. The TBK1-IRF3 axis is widely held to be the molecular pathway leading to type I interferon based signaling. Taken together, this suggests that CAG repeat RNA does not trigger interferon response. Previously, we had shown the induction of interferon-stimulated genes in 59Q-TBP-GFP expressing neuronal cells, as compared to 16Q-TBP-GFP expressing neuronal cells, but were unable to detect interferon directly. Abrogation of cell death by specific neutralizing antibodies against interferon gamma, suggested that type II interferon signaling may be involved. However, we could not establish the relative contribution of type I, II, and III signaling, if any. Here, we show that Interferon gamma and lambda genes are induced in response to 59Q-TBP, as compared to 16Q-TBP expression.

Exploring the status of interferon signaling pathways in other CAG repeat expansion disorders such as HD could reveal a central role for neuroinflammation in polyglutamine diseases. Our studies on HD mice showed an active interferon pathway signature in the brain. Although some ISGs were transcriptionally upregulated, interferon mRNA levels were not affected. This may be due to heterogeneity of the brain tissue. If the interferon-mediated effect seen in polyQ disease is neuron-specific, it may be masked in the mRNA pool of the brain tissue collected from HD mice. In such a scenario, only the highly upregulated or downregulated genes would show significant effect. Hence, further cell specific expression should be done in future studies.

In summary, CAG repeat RNA was not toxic to neuronal cells. However, such RNA when presented in the context of protein-coding transcripts, in TBP or huntingtin, consistently led to expression of RNA sensors and interferon-stimulated genes. The canonical viral RNA response pathway through IRF3 was not involved in polyQ mediated interferon signaling. Our results suggest that soluble or small aggregates may be the form in which polyglutamine protein cause neuroinflammation and apoptosis. Treatment with MG132 led to accumulation of large aggregates but surprisingly restored Stat1 expression to normal levels (Supplementary Fig. 3). We speculate that by favoring large aggregates, MG132 may have depleted the formation of soluble toxic intermediates. More detailed experiments probing the role of soluble polyglutamine are needed to reveal the mechanism by which they cause interferon release.

## Supplementary information

Supplementary Table 1

Supplementary Figure 1

Supplementary Figure 2

Supplementary Figure 3
