# Supplementary material for: Interferon mediated neuroinflammation in polyglutamine disease is not caused by RNA toxicity
Source: Cell Death Dis. 2020 Jan 2;11(1):3. doi: 10.1038/s41419-019-2193-x (PMC6952400; doi:10.1038/s41419-019-2193-x)
Supplement: Supplementary file 1 — Supplementary Table 1 [file 41419_2019_2193_MOESM1_ESM.docx]

**Supplementary Table 1: Primers used in this study**

| **Gene** | **Sequence** | |
| --- | --- | --- |
| Gapdh | FP | TGTGTCCGTCGTGGATCTGA |
|  | RP | CCTGCTTCACCACCTTCTTGA |
| Stat1 | FP | GCTGTGCCTCTGGAATGATG |
|  | RP | CGGGAGCTCTCACTGAATCT |
| Gbp3 | FP | GCATCTCCTGTGGAGCTTTCA |
|  | RP | TCACTCCCTTCCTCAGCACT |
| Usp18 | FP | CCTTGTCTGCTGCATTTCAA |
|  | RP | TTCCGTGTGTGAGCTTTCAG |
| Isg15 | FP | AAGAAGCAGATTGCCCAGAA |
|  | RP | TCGCTGCAGTTCTGTACCAC |
| Cxcl10 | FP | TCCTTGTCCTCCCTAGCTCA |
|  | RP | ATAACCCCTTGGGAAGATGG |
| Rig1 | FP | CCACCTACATCCTCAGCTACATGA |
|  | RP | TGGGCCCTTGTTGTTCTTCT |
| Lgp2 | FP | ACACAAGCCAGAGCACACAC |
|  | RP | TTGCACTGAGCGATATCCAG |
| Mda5 | FP | TCACTGATCTGCCCTCTCCT |
|  | RP | CCTTCTCGAAGCAAGTGTCC |
| Tlr3 | FP | AGCATCAAAAGAAGCCGAAA |
|  | RP | CTTGCTGAACTGCGTGATGT |
| Ifng | FP | GCTTTAACAGCAGGCCAGAC |
|  | RP | GGAAGCACCAGGTGTCAAGT |
| Ifnl3 | FP | CTCTGTCCCCAAAAGAGCTG |
|  | RP | GGAACTGCACCTCATGTCCT |
