# Supplementary figures and images for: Interferon mediated neuroinflammation in polyglutamine disease is not caused by RNA toxicity
Source: Cell Death Dis. 2020 Jan 2;11(1):3. doi: 10.1038/s41419-019-2193-x (PMC6952400; doi:10.1038/s41419-019-2193-x)

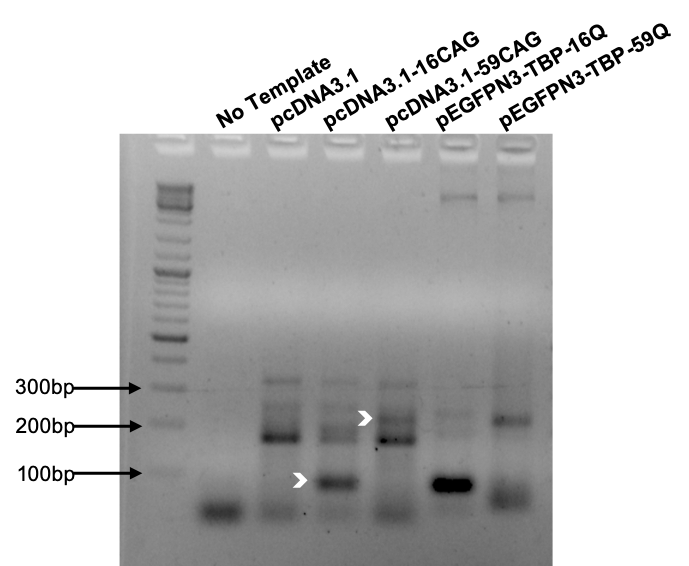

Supplement: Supplementary file 2 — Supplementary Figure 1 [file 41419_2019_2193_MOESM2_ESM.png]

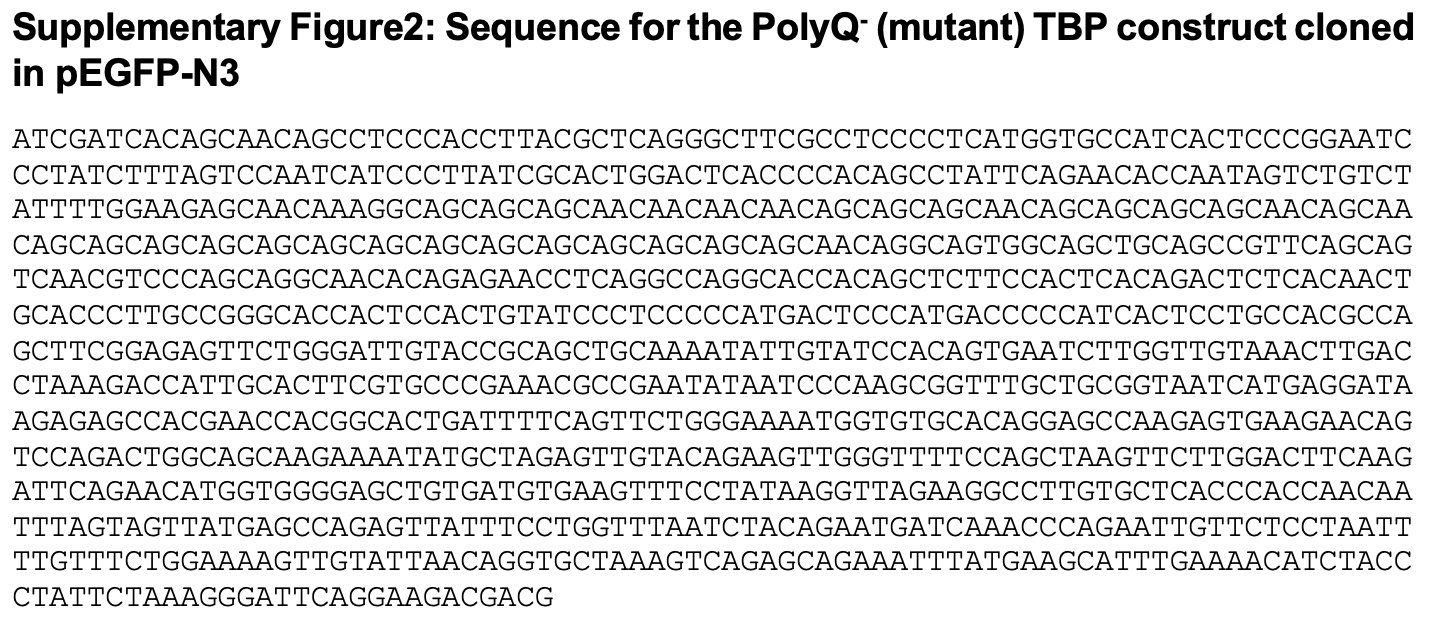

Supplement: Supplementary file 3 — Supplementary Figure 2 [file 41419_2019_2193_MOESM3_ESM.png]

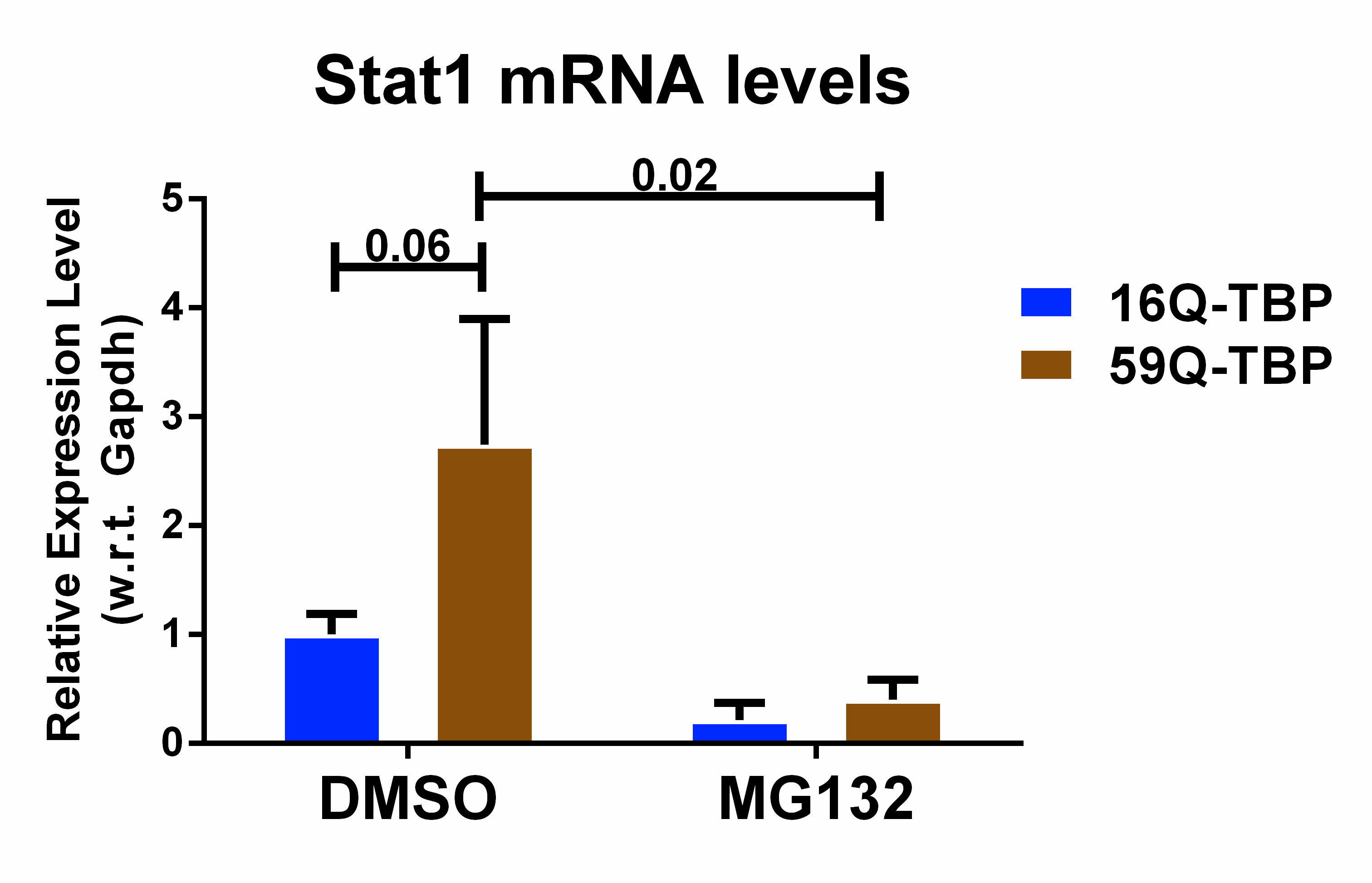

Supplement: Supplementary file 4 — Supplementary Figure 3 [file 41419_2019_2193_MOESM4_ESM.tif]
